# Reply to Lao Q and Merke DP

**DOI:** 10.1038/s41431-021-00869-y

**Published:** 2021-04-07

**Authors:** Sabina Baumgartner-Parzer, Martina Witsch-Baumgartner, Wolfgang Hoeppner

**Affiliations:** 1 Department of Internal Medicine III, Clin. Division of Endocrinology & Metabolism, Medical University of Vienna, Vienna, Austria; 2 Department of Medical Genetics, Molecular and Clinical Pharmacology, Division Human Genetics, Medical University of Innsbruck, Innsbruck, Austria; 3 Bioglobe GmbH, Hamburg, Germany

**Keywords:** Adrenal gland diseases, Genetic testing

## To the Editor:

We thank Lao Q and Merke DP for their interest in the EMQN-CAH-practice guidelines for molecular genetic testing of CAH [1] and their suggestion to include evaluation of CAH-X in routine *CYP21A2* genotyping.

We completely agree with the authors of this corresponding letter that CAH-X (CAH with a hypermobility type Ehlers–Danlos syndrome (EDS) due to pathogenic tenascin-X variants) is present in a considerable number of CAH patients [2], particularly in those who carry so-called large deletions comprising also the *TNXB* gene. In the majority of those patients CAH-X is currently probably missed.

As addressed in previous work, the use of the CAH-MLPA assay also covering exon 35 of the TNXB-gene could help in identification of a chimeric recombination between *TNXB* and its pseudogene *TNXA*, resulting in a contiguous *CYP21A2* and *TNXB* deletion associated with EDS (CAH-X).

So far, CAH-X diagnostic evaluation was not included in the CAH genotyping guideline, as from the EMQN-CAH scheme there is only very limited knowledge and experience concerning CAH-X genotyping. Comprehensive and reliable CAH-X genotyping would also include analysis of additional *TNXB* chimeras and pathogenic variants [3] not currently covered by CAH-MLPA analysis as outlined by Lao and Merke. Such CAH-X genotyping implies (i) validation as an in-house method, (ii) comprehensive understanding of the molecular background relevant for adequate interpretation of the genotyping results, (iii) the expertise of genetic counselling before and after CAH-X analysis and (iv) experience and expertise of clinical management and follow up of cardiovascular comorbidities being crucial for recognition and prevention of complications. In addition, the availability of plasma concentrations of TNX levels measured by ELISA would also be helpful.

Moreover, the respective institutions/laboratories have to define whether findings of *TNXB*-variants in the course of routine *CYP21A2* gentoyping (in case MLPA is performed) is seen as incidental finding and to which extent such findings are communicated to the individual the blood sample of whom was analyzed (who is not necessarily a CAH patient, but may also be a healthy partner/family member). This has been controversially discussed during a previous EMQN-CAH-scheme-meeting, when a sample showed a reduced signal for *TNXB*.

Another important aspect in that context is that hypermobility is frequently seen and only a minority of cases is due to CAH-X. This has to be taken into account when family members or partners of CAH-patients undergo *CYP21A2* genotyping. So, it can be foreseen that a considerable number of individuals who undergo CAH/CAH-X genotyping have signs and symptoms of hypermobility not related to pathogenic variants of *TNXB*. Thus, adequate strategies have to be developed and defined in phenotypically hypermobile individuals without CAH-X in cooperation and accordance with the respective professional societies.

We fully agree that CAH-X newborn screening would enable early diagnosis for young children before hypermobility evaluation is possible and would allow early screening for cardiac defects. In conclusion, we greatly appreciate Lao’s and Merke’s comment emphasising the importance of detecting CAH-X in patients suffering from 21-OH-defiency, as early detection and adequate therapeutic management of CAH-X will help to improve the patients’ quality of life and could prevent serious cardiac and vascular complications.

In that context it would be of major importance that further studies are performed in order to broaden the knowledge and expertise on CAH-X before including respective methods in routine diagnostic procedures.

## References

[1] Baumgartner-Parzer S, Witsch-Baumgartner M, Hoeppner W (2020). EMQN best practice guidelines for molecular genetic testing and reporting of 21-hydroxylase deficiency. Eur J Hum Genet.

[2] Merke DP, Chen W, Morissette R, Xu z, Ryzin Van, Sachdev V (2013). Tenascin-X haploinsufficiency associated with Ehlers-Danlos syndrome in patients with congenital adrenal hyperplasia. J Clin Endocrinol Metab.

[3] Lao Q, Brookner B, Merke DP (2019). High-throughput screening for CYP21A1P-TNXA/TNXB chimeric genes responsible for ehlers-danlos syndrome in patients with congenital adrenal hyperplasia. J Mol Diagn.

